# MAb PGT121 protects against mucosal SHIV challenge in macaques at concentrations corresponding to its highly potent in vitro neutralization capacity

**DOI:** 10.1186/1742-4690-9-S2-O5

**Published:** 2012-09-13

**Authors:** B Moldt, EG Rakasz, N Schultz, P Chan-Hui, K Swiderek, DI Watkins, DR Burton, P Poignard

**Affiliations:** 1 The Scripps Research Institute, La Jolla, CA, USA; 2 University of Wisconsin-Madison, Madison, WI, USA; 3 Theraclone Sciences, Inc., Seattle, WA, USA

## Background

We recently characterized several new broadly neutralizing monoclonal antibodies (bnMAbs) remarkable for their in vitro potency. A number of these bnMAbs are almost 10 fold more potent than previously isolated bnMAbs such as PG9 and VRC01 and hundred fold more potent than older prototype bnMAbs such as 2G12 and b12. Of the new antibodies, PGT121 is one of the most broad and potent, and recognizes a glycan-dependent epitope. Based on in vitro neutralization potency, we speculated that PGT121 may be protective in vivo at serum concentrations below those previously determined for other broadly neutralizing anti-HIV antibodies.

## Methods

The neutralization potency of PGT121 against SHIV _SF162P3_ was evaluated using a TZMbl-based neutralization assay. To evaluate the protective potency of PGT121 in vivo, we then designed a protection study in rhesus macaques. Animals were administered intravenously with 3 different doses of antibody (5 mg/kg, 1 mg/kg or 0.2 mg/kg) 24 hours before being vaginally challenged with a single high dose of SHIV _SF162P3_ (300 TCID50). Serum levels of antibody and viral loads were determined throughout the study.

## Results

PGT121 potently neutralizes SHIV _SF162P3_ with an IC50 of about 0.005 µg/ml. Preliminary data suggest that an administered dose of PGT121 at 1 mg/kg is sufficient to induce sterilizing immunity against a single high dose vaginal challenge of SHIV _SF162P3_. A lower 0.2 mg/kg dose appears to be partially protective.

## Conclusion

PGT121 is one of the most broad and potent neutralizing antibodies isolated to date. Here, we provide evidence that in vivo protection by PGT121 correlates with the high in vitro potency of the antibody. PGT121 can thus mediate sterilizing immunity at concentrations that are significantly lower than previously observed from passive protection studies with bnMAbs and may be readily achievable through vaccination.

